# Supplementary material for: MetaRibo-Seq measures translation in microbiomes
Source: Nat Commun. 2020 Jun 29;11:3268. doi: 10.1038/s41467-020-17081-z (PMC7324362; doi:10.1038/s41467-020-17081-z)
Supplement: Supplementary file 10 — Supplementary Data 7 [file 41467_2020_17081_MOESM10_ESM.zip › File2/Confidence_High_Taxonomy/307238_out.krona.html]

Javascript must be enabled to view this page.

members
magnitude
magnitudeUnassigned
count
unassigned
taxon
rank

307238\_out

4

2
4
superkingdom

4
phylum
1239

4
class
186801

order
4
186802

541000
family
2

genus
2
244127

species
2
1673720

SRS063518\_contig\_number\_4831SRS077392\_contig\_number\_14804

186803
family
1

658087
1
species

SRS140492\_contig\_number\_contig-100\_3429.86872

216572
1
family

459786
1
genus

1945593
species
1

SRS013965\_contig\_number\_32741
